# Heterologous prime-boost vaccination drives stromal activation and adaptive immunity against SARS-CoV-2 variants

**DOI:** 10.3389/fimmu.2025.1597417

**Published:** 2025-05-28

**Authors:** Ji Hyang Jeon, Seongryong Kim, Seo-Yeon Kim, Kwang-Soo Shin, Bongju Park, Soojeong Chang, Chang-Yuil Kang, You-Jin Kim, Jong-Eun Park, Sungsu Youk, Dokeun Kim, Jinah Yeo

**Affiliations:** ^1^ Division of Infectious Disease Vaccine Research, Center for Vaccine Research, National Institute of Health, Korea Disease Control and Prevention Agency, Cheongju, Republic of Korea; ^2^ Department of Microbiology, College of Medicine, Chungbuk National University, Cheongju, Republic of Korea; ^3^ Graduate School of Medical Science and Engineering, Korea Advanced Institute of Science and Technology (KAIST), Daejeon, Republic of Korea; ^4^ Research & Development Center, Cellid Co., Ltd., Seoul, Republic of Korea; ^5^ Biomedical Research Institute, Chungbuk National University Hospital, Cheongju, Republic of Korea; ^6^ Center for Vaccine Research, National Institute of Health, Korea Disease Control and Prevention Agency, Cheongju, Republic of Korea

**Keywords:** COVID-19, heterologous vaccination, cross immunity, mRNA vaccine, adenoviral vaccine, single cell transcriptional analysis, immune response

## Abstract

Heterologous vaccination strategies have shown superior efficacy over homologous regimens in clinical studies, but the underlying immunological mechanisms remain incompletely understood. Using a mouse model, we investigated the immune responses induced by heterologous prime-boost vaccination with adenoviral and mRNA vaccines. Heterologous vaccination (adenoviral prime, mRNA boost) elicited higher neutralizing antibody titers and stronger CD8^+^ T cell responses against Delta and Omicron-BA.5 variants compared to homologous regimens. Single-cell transcriptomic analysis of injection-site tissues revealed that adenoviral priming induced minimal changes in cellular composition but established a pre-conditioned innate immune environment. This effect was further amplified upon mRNA boosting, particularly through fibroblast-driven chemokine responses that promoted immune cell recruitment. These findings suggest that adenoviral priming enhances local immune activation upon boosting, contributing to the heightened adaptive immune response observed in heterologous vaccination. This study provides mechanistic insights into the immunological effects of heterologous prime-boost strategies against SARS-CoV-2 variants.

## Introduction

Since 2019, severe acute respiratory syndrome coronavirus 2 (SARS-CoV-2) caused the global pandemic known as coronavirus disease 2019 (COVID-19), which posed substantial public health challenges. Vaccination has been one of the most effective strategies against SARS-CoV-2, with various platforms, such as adenovirus-based vectors, mRNA, and protein subunit vaccines, developed and approved (1, 2). The unprecedented nature of the COVID-19 pandemic underscored the urgent need for diversifying vaccine platforms, which has subsequently driven the development and clinical testing of multiple vaccine technologies (3, 4).

The continued endemicity and zoonotic nature of SARS-CoV-2 have contributed to the emergence of new variants (5). Mutations in the spike protein, the major antigen of SARS-CoV-2, necessitate periodic updates of vaccine strains, posing a significant burden on public health systems and challenging the ability of vaccine manufacturers to maintain their long-term effectiveness and efficacy (6, 7). As of April 2025, WHO lists JN.1 as the dominant global variant and monitors its sub-lineages, including KP.3 (8).

During the COVID-19 pandemic, substantial international interest in heterologous prime-boost vaccination strategies emerged to deal with potential supply chain disruptions or shortages that could otherwise slow down vaccine distribution and herd immunity (2, 9–11). Clinical observations have demonstrated that heterologous boosters, regardless of whether the combination involves whole inactivated vaccines, subunit vaccines, mRNA vaccines, or adenovirus-vectored vaccines, confer superior serum neutralization titer (12–15) and cellular immune response (16–18) compared to homologous boosters. Compared to homologous vaccination, heterologous vaccination significantly increased spike-specific CD8^+^ T cells, and neutralizing antibody titers increased 4–20 times with homologous vaccination, whereas heterologous vaccination reached a 6–73-fold increase (19). Animal experimental data have also confirmed marked increases in both T cell immune responses and neutralizing antibody titers (20, 21). However, the immunological mechanisms underlying the observed increase in the immune response remain to be elucidated.

Particularly, adenovirus-based vaccines may contribute to heterologous efficacy through trained immunity (22–24). Trained immunity refers to a memory-like state within the innate immune system in which innate cells, especially monocytes and macrophages, are epigenetically and metabolically reprogrammed to respond more robustly to future challenges, even from unrelated pathogens (25). Several studies have demonstrated that adenoviral vaccines induce prolonged activation of monocytes, resulting in enhanced cytokine production and antigen presentation capabilities for up to 3 months post-vaccination (22, 23, 26). This phenomenon, marked by increased expression of glycolytic enzymes and inflammatory cytokines, primes the immune system to respond more effectively to subsequent exposure, potentially boosting both innate and adaptive immunity in a heterologous prime-boost setting (27–29).

To investigate the immunological mechanisms underlying heterologous prime-boost vaccination with adenovirus-vectored and mRNA COVID-19 vaccines, we conducted a study using a mouse model, allowing direct comparison of vaccine platforms under identical conditions. Humoral and cellular immune responses were compared using serological assays and antigen-specific T-cell responses against SARS-CoV-2 variants, including Delta and Omicron-BA.5. In detail, single-cell RNA sequencing of injection site tissues allowed for the observation of changes in immune cell infiltration and associated inflammatory responses, offering insights into the distinct immune reactions elicited by different vaccine types and vaccination regimens. To our knowledge, this is the first study to systematically confirm the immunological benefits of heterologous vaccination (adenoviral prime, mRNA boost) in a mouse model, complementing previous clinical observations.

## Materials and methods

### Mice and immunization

Female BALB/c mice aged 4–6 weeks were acquired from Samtako Bio (Korea). The mice were housed and bred in the Animal Biosafety Level 2 facility of the National Institute of Health, South Korea. Vaccines were administered to the hind limb muscles. The mice were immunized under one of the following conditions: two shots of either PBS, empty LNP (equivalent to the lipid content of 5 μg mRNA-LNP), 5 μg mRNA-LNP, or 2 × 10^8^ viral particles of adenovirus-vector vaccine. Additionally, a combination regimen was tested, consisting of a priming dose with 2 × 10^8^ adenovirus-vector vaccine followed by a boost dose with 5 μg mRNA-LNP. All prime and booster vaccines were administered 3 weeks apart. For collection of samples, mice were anesthetized by intraperitoneal administration of 10 mg/kg Rompun and 100 mg/kg Ketamine. Blood was obtained by facial vein puncture under anesthesia. Euthanasia was performed by CO_2_ inhalation at a flow rate of 30% of the chamber volume per minute, ensuring a gradual displacement to minimize animal distress. Following euthanasia, spleens were collected from the mice. To evaluate cellular and humoral responses, six spleen and four blood samples from each group were collected 3 weeks after booster vaccination. No significant changes in weight or behavior were observed in the mice following any of vaccination regimens. For single-cell transcriptomic analysis of the injection site, one muscle sample from each group was collected 16 h after both prime and booster vaccinations. The animal experimental protocol used in this study was reviewed and approved by the Institutional Animal Care and Use Committee of the Korea Centers for Disease Control and Prevention (KDCA-IACUC-22-004).

### COVID-19 mRNA vaccine and adenovirus-vector vaccine

The SARS-CoV-2 mRNA vaccine used in this study encodes the Spike (S) antigen derived from SARS-CoV-2 isolate 2019-nCoV/USA-WA1/2020 (GenBank: MN985325). The mRNA vaccine was prepared using a previously established method (30). Briefly, a human codon-optimized S sequence with two proline substitutions (K986P/V987P) was synthesized and cloned into an mRNA production plasmid. A linearized DNA template containing an open reading frame flanked by 5′ and 3′ untranslated regions and 120-nucleotide poly(A) tails was produced by PCR amplification. mRNAs were synthesized *in vitro* using T7 RNA polymerase–mediated transcription with complete replacement of uridine by N1-methyl-pseudouridine-5′-triphosphate (m1Ψ). Capping of the *in vitro*–transcribed mRNAs was performed co-transcriptionally using the trinucleotide cap1 analog (m7(3’OMeG)(5’)ppp(5’)2’OMeA)G). LNPs were prepared using a NanoAssembly Benchtop Instrument (Precision Nanosystems Inc.). Ionizable lipids (SM-102, 06040008800, SINOPEG), cholesterol (C3045-100G, Sigma-Aldrich), distearoylphosphatidylcholine (DSPC, 850365P, Avanti), and PEG-lipid (DMG-PEG, 880151P-1g, Avanti) were dissolved in ethanol, and the mRNA was diluted in 10 mM citrate (pH 3). The molar ratio of lipid components was SM-102:DSPC: Cholesterol : PEG= 50:10:38.5:1.5. The final ionizable lipid:RNA weight ratio was 10:1, and the final volume ratio was 1:3. LNPs were formulated by microfluidic mixing of the prepared solutions at a flow rate of 12 mL/min. The resulting LNPs were diluted in a 40-fold volume of 1× PBS and concentrated via ultrafiltration.

The adenovirus-vectored vaccine (AdCLD-CoV19-1) was provided by Cellid Co (31). All replication-incompetent recombinant adenovirus-vector vaccines used in this study had the E1 and E3 genes of the adenovirus deleted, and the E4orf6 gene was rearranged to the E1 region to minimize the incidence of replication-competent adenovirus. The fiber, which is the cell receptor-binding site of adenovirus serotype 5, was replaced with the knob of adenovirus serotype 35. AdCLD-CoV19–1 is driven by the CMV promoter and was constructed using the SARS-CoV-2 codon-optimized spike protein gene, pCMV3-SARS-CoV-2 (GenBank ID: QHD43416.1) (Sino Biological, Beijing, China), located in the E1 deficient region. The transmembrane and cytoplasmic domains of the spike protein were truncated for extracellular secretion. The furin cleavage site was mutated to GGGGS.

### Cell and virus harvest

Vero E6 cells (ATCC, CRL-1586) were cultured in Dulbecco’s modified Eagle’s medium supplemented with 10% heat-inactivated fetal bovine serum (FBS) and 1% penicillin/streptomycin (P/S). The cells were cultured at 37°C with 5% CO_2_ in an incubator. The SARS-CoV-2 strains used in this study as infectious viruses, including ancestral D614-like virus (D614), Delta, BA.5 variants (NCCP43326, NCCP43390, NCCP43426), were provided by the National Culture Collection for Pathogens (NCCP). The strains were cultured and titrated in Vero E6 medium using plaque-forming units (PFU). All experiments involving infectious viruses were conducted in a biosafety level 3 facility in accordance with recommended safety precautions.

### Enzyme-linked immunosorbent assay

To analyze SARS-CoV-2 specific IgG levels, each well of a 96-well plate (442404, Thermo Scientific) was coated with 50 ng of SARS-CoV-2 spike extracellular domain protein (40589-V08B1, Sino Biological) in 50 μL at 4°C overnight. The wells were blocked with 50 μL PBS containing 10% skimmed milk at 37°C for 1 h, followed by washing twice with PBS containing 1% Tween-20 (0.02% PBST; BP065, BioSolution). Serum from the immunized mice was serially diluted (three-fold) starting at 1:100 in PBS containing 3% skimmed milk and added to the virus-coated wells. Each sample was tested in duplicate and incubated at 37°C for 2 h. After washing three times with 0.02% PBST, horseradish peroxidase (HRP)-conjugated anti-mouse IgM (PA1-84383, Invitrogen), IgG (62-6520, Invitrogen), IgG1 (PA1-74421, Invitrogen), and IgG2a (M32207, Invitrogen) secondary antibodies were diluted 1:5000, added to the wells, and incubated at 37°C for 1 h. The wells were washed five times with 0.02% PBST, and the TMB substrate (T3550-050, GenDEPOT) was added to the wells and incubated at room temperature for 10 min. The reaction between TMB and HRP-conjugated anti-mouse IgG was stopped by adding the TMB stop solution (T4550-005, GenDEPOT). The absorbance was measured at 450 nm using an ELISA reader (SpectraMax i3x Molecular Device, USA), and the endpoint titer was calculated. The endpoint titer was determined as 6X the average absorbance of the negative control at 450 nm.

### Plaque reduction neutralization assay

PRNT was used to analyze the neutralizing antibodies against the mRNA vaccine. The PRNT titer was determined as the highest serum dilution that reduced the number of plaques by more than 50% compared to the number of plaques in the absence of test serum. Serum samples at 3 weeks post-booster vaccination were heat-inactivated at 56°C for 30 min before use. The prepared serum samples from immunized mice were serially diluted in serum-free medium from 1:20 to 1:10240. The serially diluted serum was mixed with an equal volume of diluted virus (50 PFU per well) to prepare the virus-serum mixture, and the mixture was incubated at 37°C incubator with 5% CO_2_ for 1 h. Vero E6 was seeded in 12-well plates (2 × 10^5^ cells/well) and infected with the virus-serum mixture, then incubated at 37°C incubator for 1 h. After viral adsorption, the cells were overlaid with 1 mL of 1.2% agarose overlay medium. For the D614 and BA.5 variants, incubation was conducted for 2 days, while for the Delta variants, incubation was conducted for 3 days in a 37°C incubator. The overlay medium was removed from the 12 well plate, fixed and stained with crystal violet solution (final concentration: crystal violet; 0.07%, 37% formaldehyde; 8%, ethanol; 5%). After removal and drying at room temperature, the plaques were counted to calculate the PRNT_50_ titer.

### Enzyme-linked immunospot assay

An ELISpot assay was conducted to verify cellular immune responses. IFN-γ-secreting cells were detected using the Mouse IFN-γ ELISpot Kit (XEL485, R&D System). Experiments were performed according to the manufacturer’s instructions. At 3 weeks post-booster vaccination, splenocytes were dissociated by mechanical disruption using a GentleMAX machine (Miltenyl Biotec, Germany). The cells were filtered through a 40 µm pore size strainer, and red blood cells were removed using ACK lysis buffer (BP10-548E, Lonza). The splenocytes were suspended in RPMI containing 10% FBS and 1% P/S and seeded at 2.5 × 10^5^ cells per 100 μL into 96-well polyvinylidene fluoride-based microplates coated with a mouse IFN-γ-specific monoclonal antibody (890894, R&D System). Splenocytes were stimulated with 100 ng/well of the SARS-CoV-2 spike glycoprotein peptide pool: D614 (RP30020, GenScript), Delta (RP30033, GenScript), and BA.5-Omicron (RP30020, GenScript). The assay included a negative control with only medium and a positive control with a cell-stimulation cocktail (00-4970-93; eBioscience™). Cells were incubated in a 37°C incubator for 18−24 h. After incubation, cells were removed from the plates, washed with a washing buffer (895308, R&D Systems), and treated with a biotinylated monoclonal antibody specific for mouse IFN-γ (890895, R&D Systems) for 2 h at room temperature. After washing, the plates were incubated with alkaline phosphatase-conjugated streptavidin (895358; R&D Systems) for 2 h. Spots were developed using a 5-bromo-4-chloro-3-indolyl phosphate/nitro blue tetrazolium (BCIP/NBT) substrate (895867, R&D Systems) for 1 h at room temperature. Spots were counted using a Cellular Technology Limited ImmunoSpot analyzer (S6 Universal M2 v.7.0 Immunospot, USA).

### Flow cytometry analysis

Spleens were collected 42 days after priming (21 days after boost), and the cells were mechanically dissociated using a GentleMAX machine. The cells were filtered through a 40 µm strainer, and red blood cells were removed using ACK lysis buffer. The cells were suspended in RPMI containing 10% FBS and 1% P/S and seeded at 2.5 × 10^5^ cells per 100 µL in a 96-well U-bottom plate. The cells were stimulated with 100 ng/well of SARS-CoV-2 spike glycoprotein crude D614 (RP30020, GenScript), Delta (RP30033, GenScript), or BA.5-Omicron (RP30020, GenScript) in complete RPMI 1640 medium. GolgiPlug (51-2301KZ, BD) was added to each well, and the cells were incubated at 37°C for 18−24 h. Stimulated cells were collected in tubes and washed with PBS. For Live/Dead staining, the cells were stained with Aqua Fluorescent Reactive Dye (1:300 dilution, L34965, Invitrogen) and incubated at 4°C for 30 min. After washing, the cells were stained with the following surface marker antibodies: anti-CD3 (1:200 dilution; APC/Cyanine7; 100222, BioLegend), anti-CD8 (1:200 dilution; PerCP/Cyanine5.5; 100734, BioLegend), anti-CD4 (1:200 dilution; Brilliant Violet 421™; 100437, BioLegend) and added to the cells in flow cytometry staining buffer (00-4222-26, eBioscience) and incubated at 4°C for 30 min. For intracellular cytokine staining, the cells were washed with Cytofix/Cytoperm solution (51-2090KZ, BD) containing staining buffer and incubated at 4°C for 1 h. The cells were then washed twice with 1X perm/wash buffer (51–2091 KZ, BD). Anti-IFN-γ antibody (1:100 dilution, APC, 554413, BD), and anti-TNF-α antibody (1:100 dilution, PE-Cy™7, 557644, BD) were added to the cells and incubated at 4°C for 1 h. Finally, the cells were washed with 1X Perm/Wash buffer and resuspended in flow cytometry staining buffer. Data were collected on a CytoFLEX LX flow cytometer (Beckman Coulter) and analyzed using FlowJo software v10.

### Preparation of single-cell RNA sequencing samples

Muscles at the injection site were dissected and washed with 1X PBS. For tissue digestion, the samples were incubated with Type II collagenase (LS004177, Worthington) at 37°C for 40 min while shaking. To improve cell yield and quality, the cells were further incubated with Type II collagenase and Dispase II (04942078001, Roche) at 37°C for an additional 20 min while shaking. To break down the remaining muscle fragments, the samples were passed several times through a 5 mL syringe fitted with an 18-gauge needle. Cells were filtered through a 40 μm strainer, rinsed with wash buffer, and centrifuged at 525 g for 5 min at room temperature. To remove the red blood cells (RBCs), the cell pellet was treated with RBC lysis buffer for 3 min at room temperature. After RBC lysis, the cells were washed and resuspended in 10 mL of complete RPMI medium. Cells were filtered using Flowmi Cell Strainers with a 40 μm pore size (136800040, Merck) to completely remove debris.

Similar to the muscle preparation, the draining iliac and inguinal lymph nodes near the injection site were collected at the same time points. Lymph nodes were digested with collagenase D (11088866001, Roche) at 37°C for 20 min. Samples were placed on a 40 μm strainer and mashed to create a single-cell suspension. After adding the RBC lysis buffer and waiting for 3 min, the cells were washed with RPMI medium. The cells were resuspended in 10 mL of complete RPMI and counted using a hemocytometer.

To generate single-cell suspensions for scRNA-seq, cell pellets were resuspended up to 10^6^ cells per milliliter of 0.04% bovine serum albumin (130-091-376, Miltenyi Biotec). scRNA-seq libraries were prepared using the Chromium Next GEM Single Cell 3’ Library Kit v3.1 (1000268, 10x Genomics). Single-cell gel beads in emulsions were created with a Chromium Controller (100171, 10x Genomics), with a targeted cell recovery of 10,000 cells. Following reverse transcription, cDNAs were pre-amplified with 11 reaction cycles. Fragmentation, adaptor ligation, and SPRI select processes were conducted according to the manufacturer’s guidelines. Fragment sizes of the constructed libraries were measured with Bioanalyzer (2100, Agilent Technologies). Sequencing of the constructed libraries was performed with Novaseq 6000 (Illumina), with a per-sample library size of approximately 100 Gb.

### Single-cell RNA-seq data analysis

Single-cell count matrices were generated using the CellRanger (v.6.0.2) pipeline, using mm10 reference, and downstream analysis was performed based on the Scanpy (32) pipeline (v.1.9.1). For quality control, low-quality cells meeting any one of the following criteria were excluded from downstream analysis (1): UMI gene count <1000, (2) number of genes detected <500, (3) number of genes detected >7000, and (4) fraction of mitochondrial genes >10%. Additionally, predicted doublets from Scrublet (33) (v.0.2.3) simulations also were removed. The transcript counts were normalized (10,000 counts per cell) and log-transformed (log1p). Highly variable genes (n=2,619) were selected with the highly_variable_genes function in the Scanpy package. Subsequently, the expression matrix of the highly variable genes was standardized using the scale function of the preprocessing module in the Scanpy package. PCA (number of PCs = 50) was conducted based on the scaled expression values of highly variable genes. Neighborhood graphs of the cells were constructed with the BBKNN (34), and we used the UMAP function in the tool module of the Scanpy package to calculate UMAP embeddings. For detection of spike mRNA component of the mRNA vaccine and the adenoviral vaccine transcripts, raw sequence files were aligned to a custom-built references containing information about mRNA and adenoviral vector construct using CellRanger (v.6.0.2), and cut-off values of mRNA and adenoviral component detection were set to 10 and 1, respectively.

### Differential abundance testing and differentially expressed gene analysis

Differential cell composition testing was conducted with scCODA (35), a Bayesian model for differential cell composition analysis in single-cell transcriptome data. The cut-off value for the false discovery rate (FDR) was set to 0.05, and only the log-fold changes below the FDR cut-off value were used. DEG analysis was performed using the rank_genes_groups function in Scanpy (v.1.9.1) package. Log-normalized counts of the cells were compared using the Wilcoxon rank-sum method, and *P* values were corrected with the Benjamini-Hochberg method. Genes with adjusted *P* < 0.05 and log_2_FC > 1 were considered as DEGs.

### DEG vector analysis

We conducted DEG vector analysis as described previously report (30). Briefly, using the saline-injected sample as a reference, we conducted DEG analysis on each cell type in each treatment sample. DEG vectors were constructed in two steps. First, we fetched log-fold change (logFC) vectors and adjusted *P* value vectors of genes from the DEG results, which is generated by rank_genes_groups from ‘tl’ module in the Scanpy package. Next, for the genes with adjusted *P* > 0.05, values in the logFC vectors were replaced with 0 to ensure robustness of the logFC values. The DEG vectors were projected on previously identified axes of transcriptional responses (30). The projection matrix, which consists of coefficients fitted in the previous study, was used for the PC projection of the DEG vectors in this study.

### Gene set enrichment analysis

Gene set enrichment analysis was performed using the enrich function in the GSEApy (36) package (v.1.0.4). For upregulated or downregulated DEGs, we used genes with adjusted *P* < 0.05 in each direction. Gene set used for analysis with GSEA was the Molecular Signature Database (MSigDB) hallmark collection (37). To calculate gene set signature scores, we calculated average expression (log-transformed normalized counts) of genes included in each gene set.

### Statistical analysis

Statistical analyses were performed using GraphPad Prism software (version 10.0; GraphPad Software Inc., San Diego, CA, USA). Data for grouped pairs were analyzed using a two-way analysis of variance test and expressed as the standard deviation of independent experiments. Differences were considered statistically significant at a *P*-values < 0.05.

## Results

### Heterologous vaccination enhances neutralizing antibodies against SARS-CoV-2 variants in BALB/c mice

**Figure 1:**
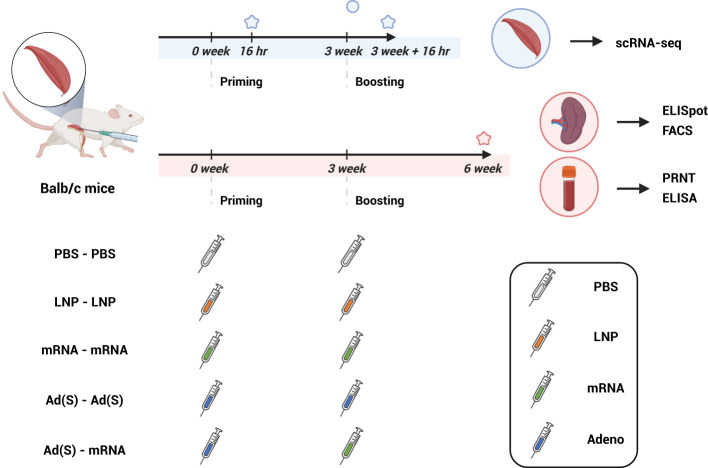
Experimental design and sampling schedule (created with BioRender.com). BALB/c mice were intramuscularly immunized with two doses of phosphate-buffered saline (PBS), lipid nanoparticle (LNP), or 5 μg of mRNA vaccine (mRNA) and 2 x 10^8^ adenovirus-vector vaccine (Ad(S)) for the first dose, followed by 5 μg of mRNA vaccine for the second dose. Muscle tissues at the injection site used for single-cell RNA sequencing were sampled 16 h after the first vaccination and 16 h after the booster vaccination. Spleen and blood samples were collected 3 weeks after the boost vaccination and were evaluated to confirm cellular response (ELISpot, FACS) and antigen-specific humoral response (PRNT, ELISA), respectively. ELISpot, enzyme-linked immunospot; ELISA, enzyme-linked immunosorbent assay; PRNT, plaque reduction neutralization test.

**Figure 2:**
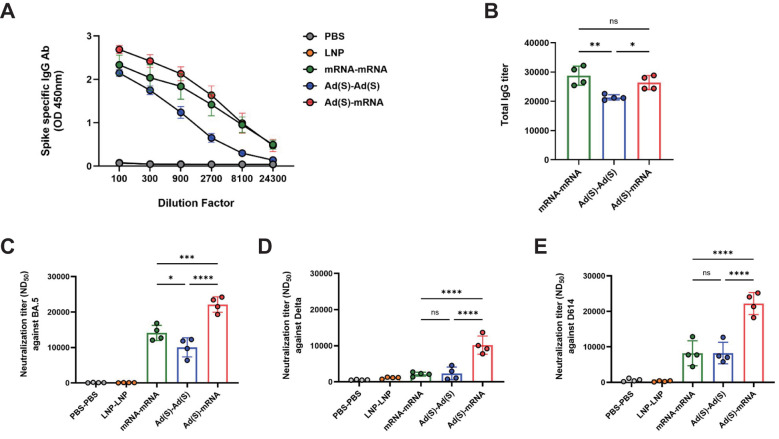
Heterologous vaccination enhances humoral immunity in BALB/c mice. **(A)**, Kinetics of severe acute respiratory syndrome coronavirus 2 (SARS-CoV-2) spike specific binding antibody responses against total immunoglobulin G (IgG). The blood samples of vaccinated mice were collected 3 weeks after the booster vaccination. **(B)**, Sera of vaccinated mice were analyzed for SARS-CoV-2 specific total IgG using enzyme-linked immunosorbent assay (ELISA). **(C–E)**, The neutralizing antibody titers in the serum of the vaccinated mice were analyzed via a plaque reduction neutralization test (PRNT) using the SARS-CoV-2 virus variants against **(C)** D614, **(D)** Delta, and **(E)** BA.5. *P*-values were determined using two-way analysis of variance (ANOVA) and Tukey’s multiple comparison test. * *P* < 0.05, ** *P* < 0.01, *** *P* < 0.001, **** *P* < 0.0001.

In this study, we investigated the immunogenicity of SARS-CoV-2 spike (from the original strain, D614) vaccines delivered using adenoviral vectors and lipid nanoparticle (LNP) mRNA platforms in homologous and heterologous prime-boost formats (**Figure 1**). To assess the binding and neutralizing antibody responses, ELISA and PRNT assays were conducted on sera collected 3 weeks after the booster shot. Spike-specific immunoglobulin M (IgM) levels remained low and were comparable across all groups, with a response in the empty LNP group likely reflecting non-specific activation (**Supplementary Figure 1**). Spike-specific total IgG titers were higher in the heterologous vaccination group (Ad-mRNA) than in the homologous vaccination group (Ad-Ad), whereas there was no significant difference in titers between the homologous mRNA-mRNA and Ad-mRNA groups (**Figures 2A**). This indicates that antigen-specific IgG antibody levels, which undergo isotype class switching from IgM to IgG, were not compromised in the heterologous Ad-mRNA vaccination group compared to the homologous mRNA-mRNA vaccination group.

Neutralizing antibody (nAb) levels were measured using the PRNT assay against the D614 (**Figure 2**), Delta (**Figure 2**), and Omicron-BA.5 (**Figure 2**) variants. For the D614 and Delta variants, no significant differences in nAb titers were observed between the homologous mRNA (mRNA-mRNA) and adeno-vaccine groups (Ad-Ad), whereas the heterologous vaccination group showed significantly higher titers. For the Omicron-BA.5 variant, the nAb titers were highest in the heterologous group, followed by the homologous mRNA and homologous adeno groups. These findings suggest that the heterologous vaccination regimen induces robust nAbs across SARS-CoV-2 variants compared with homologous regimens.

### Heterologous vaccination induces robust antigen-specific T-cell responses against SARS-CoV-2 variants

**Figure 3:**
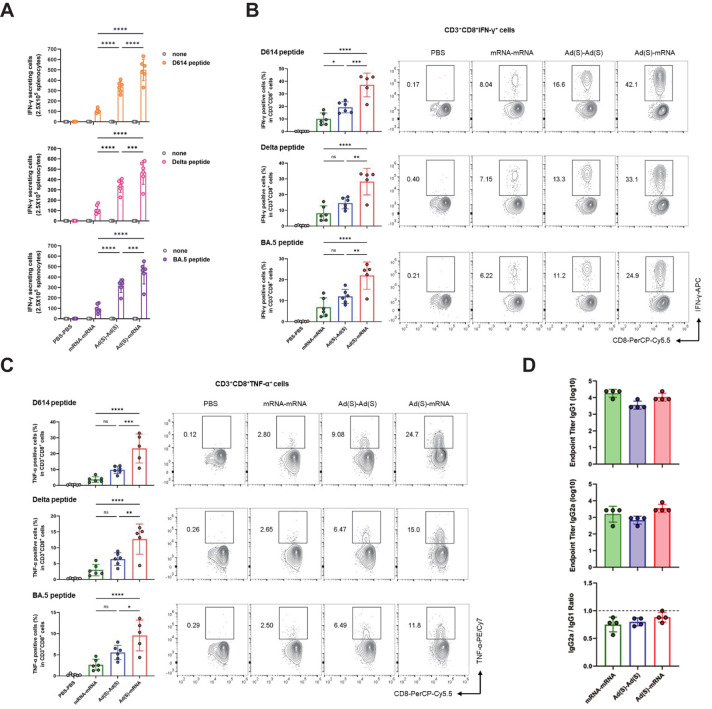
Heterologous vaccination induces robust CD8^+^ T cell responses in BALB/c mice. **(A)**, IFN-γ ELISpot analysis was measured for T cell responses. Splenocytes from immunized mice were collected 3 weeks after the final vaccination and stimulated with D614, Delta, and BA.5 spike glycoprotein peptide pool and compared to those in unstimulated cells. Sample wells with spots were measured using a CTL Immunospot reader. **(B, C)**, Flow cytometry analysis of intracellular cytokines **(B)** IFN-γ, **(C)** TNF-α. Antigen-specific CD8 T cells were obtained from mouse spleen samples on day 42 post-vaccination. The IFN-γ, TNF-α CD3^+^CD8^+^ cells were stimulated with D614, Delta, and BA.5 spike glycoprotein peptide pool. **(D)**, The IgG subtypes in the sera of vaccinated mice were assessed by ELISA for SARS-CoV-2 spike-specific IgG1 and IgG2a. Endpoint titers and endpoint titer ratios of IgG2a to IgG1 were calculated. *P*-values were determined using two-way ANOVA and Tukey’s multiple comparison test (ns: nonsignificant, * *P* < 0.05, ** *P* < 0.01, *** *P* < 0.001, **** *P* < 0.0001).

Cellular immune responses were assessed using ELISpot and flow cytometry on splenocytes from vaccinated mice 3 weeks after booster vaccination. For the ELISpot analysis, the splenocytes were stimulated with a D614, Delta, or BA.5 strain-based SARS-CoV-2-spike glycoprotein peptide pool, and then interferon gamma (IFN-γ)-producing T cells were detected. IFN-γ production in responses to stimulation with D614, Delta, and Omicron BA.5 variant peptides was consistently higher in the heterologous group than in the homologous groups (**Figure 3**), suggesting a strong T-cell-mediated immune response across all tested variants. Consistent with previous clinical studies, the adenovirus vaccine group (Ad-Ad) showed higher cellular immunogenicity than the mRNA vaccine group (mRNA-mRNA) (38, 39).

Flow cytometry analysis further confirmed that the heterologous group exhibited elevated levels of antigen-specific CD8 T cells producing IFN-γ and tumor necrosis factor-alpha (TNF-α) (**Figures 3B**). Additionally, the frequency of IFN-γ and TNF-α double-positive CD8 T cells was significantly higher in the heterologous group, indicating robust antigen-specific activation and cytotoxic immune responses (**Supplementary Figure 2A**). In contrast, antigen-specific CD4 T cells producing IFN-γ and TNF-α showed no statistically significant differences between heterologous vaccination (Ad-mRNA) and homologous vaccination (mRNA-mRNA or Ad-Ad) for each variant peptide (**Supplementary Figures 2B, C**), although a trend toward increased cytokine co-expression was observed in the heterologous group (**Supplementary Figure 2D**). These findings suggest a robust enhancement of CD8 T cell responses, while CD4 T cell activation showed an upward trend that did not reach statistical significance. The analysis of the spike-specific IgG subtypes, IgG1 and IgG2a, showed an IgG2a/IgG1 ratio close to 1 across all three vaccine groups (**Figure 3**). This observation indicates that the three vaccination regimens maintain a similar Th1/Th2 immune response balance, supporting both humoral and cellular immunity (40, 41).

Overall, these results demonstrate that while the homologous and heterologous regimen exhibit comparable Th1/Th2 immune response balance, the heterologous vaccination generally induced stronger cellular and humoral immunity compared to the homologous vaccination. This enhanced immune response aided in broader neutralization capacity against SARS-CoV-2 variants, underscoring the efficacy of the heterologous regimen in inducing robust and balanced immunity.

### Single-cell analysis reveals distinct injection-site immune responses to mRNA and adenoviral vector vaccination

**Figure 4:**
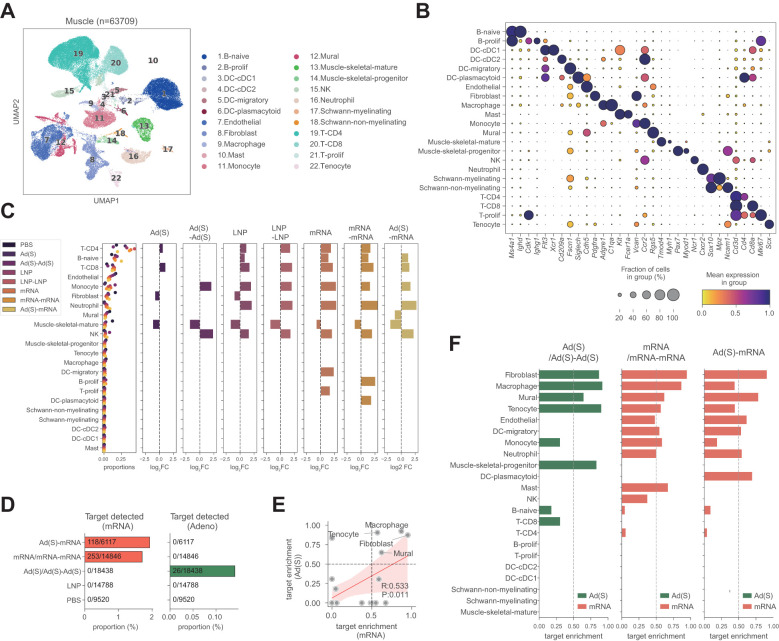
Single-cell transcriptomic analysis reveals distinct injection-site immune responses. **(A)**, UMAP visualization of injection site cells across all vaccine groups. Clusters are numbered on the plot, with corresponding cell types annotated in the accompanying panel based on canonical marker expression. **(B)**, Major cell type markers are presented in a dot plot. Colors indicate min-max normalized average expression levels in each cell type, and dot sizes represent the ratio of cells expressing marker genes in each cell type. **(C)**, Proportion of cell types according to the injection type. Proportions of each cell type are presented in the left panel, and the right panels show differential abundances (log2 fold changes) of cell types in each injection type (compared to the PBS injection condition). **(D)**, Target molecule detection rate across samples. The mRNA and adenoviral vaccines in the homologous vaccination group represent the combined results of prime and boost, while the heterologous vaccination group includes results of the boosting of adenovirus vaccination with the mRNA vaccine. For each group, spike-positive cell counts and total cell counts are shown on the bars. **(E)**, Target enrichment profile across cell types. **(F)**, Comparison of target enrichment pattern in mRNA and adenoviral injection condition.

Innate immune responses are critical for shaping adaptive immunity, as shown in recent studies (42–45), including our previous work highlighting stromal activation at the injection site (30). To further investigate the innate immune dynamics underlying these enhanced responses, we analyzed the injection site microenvironment using single-cell transcriptomics [**Figures 4A**; LNP and LNP-mRNA single-cell sequencing data from the injection site atlas study (30)]. Both the mRNA vaccine and empty LNP injection induced substantial changes in innate and adaptive immune cells, notably monocytes and neutrophils. In contrast, the adenoviral vector vaccination maintained a relatively stable immune cell composition after the first administration, suggesting a less reactive immune environment (**Figure 4**). After the second adenoviral vector vaccination (Ad-Ad), an increased monocyte count was observed, similar to the response observed with the LNP-only and mRNA vaccinations. However, unlike LNP-only and mRNA vaccinations, no changes were observed in adaptive immune cells, including CD4^+^ T cell, CD8^+^ T cell, or B cell populations, following adenoviral vector boost.

For spike mRNA detection, the mRNA vaccine showed a much higher detection rate (1–2% of counted cells) than the adenoviral vector vaccine (~0.2%) (**Figure 4**). This difference arises from distinct mechanisms of action: mRNA vaccines deliver spike mRNA directly to the cytoplasm for immediate translation, whereas adenoviral vectors require transcription from DNA, leading to slower and less direct spike mRNA expression. Regardless of this difference in detection rates, spike mRNA was predominantly enriched in fibroblasts and macrophages at the injection sites of both vaccines (**Figures 4E**). This indicates that these cell types may play a crucial role in the local immune response and spike protein expression, regardless of the vaccine platform.

### Adenoviral priming establishes an innate immune environment, enhanced by mRNA boosting

**Figure 5:**
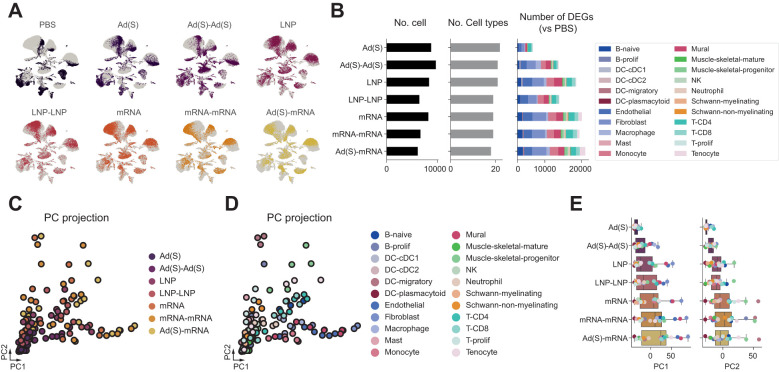
Adenoviral priming conditions the injection site for enhanced immune activation upon boosting. **(A)**, Single-cell transcriptomic landscape of injection sites according to the injection conditions. **(B)**, Number of differentially expressed genes (DEGs) across cell types in each injection condition (right). Total number of cells analyzed per group after quality control filtering (left). Number of distinct cell types identified in each group (middle). Number of differentially expressed genes (DEGs) across cell types in each injection condition (right). **(C)**, DEG vectors projected on principal component (PC) axes of injection site responses. Each dot represents the DEG vector of cell types in each injection condition. **(D)**, PC projections of DEG vectors according to the injection conditions.

Single-cell transcriptomic analysis demonstrated that the adenoviral vector booster vaccination triggered significantly stronger transcriptional changes than the first shot (**Figure 5**). To compare the immune responses between prime and boost vaccinations, we analyzed differentially expressed genes (DEGs) across various cell types. The adenoviral vector boost shot induced a substantially larger number of DEGs than the prime dose, with fibroblasts and endothelial cells showing the most pronounced changes (**Figure 5**).

Principal component analysis (PCA) of the DEG vectors further highlighted distinct response patterns. The PC1 axis, which reflects stromal cell activity (including fibroblasts, endothelial cells, and mural cells), showed a marked increase following the adenoviral boost compared to the prime. Similarly, the PC2 axis, associated with migratory dendritic cell (mDC) activity, showed notable amplification after the adenoviral boost (**Figures 5C**). These findings indicate that the adenoviral booster vaccination effectively enhances stromal and dendritic cell-mediated immune responses compared to the initial priming dose, suggesting robust local activation of innate immune pathways. However, as anticipated from the comparison between a single dose of adenoviral vector or mRNA, the magnitude of these responses was notably lower than the heterologous regimen (Ad-mRNA). Interestingly, the heterologous vaccination appeared to enhanced responses along the PC1 axis compared with the homologous mRNA regimen, driven by the elevated PC1 axis activity in fibroblasts and other stromal cell types. While the average PC2 axis responses did not increase in the heterologous group, mDC activity along this axis was observed to be the highest, indicating role for DCs in the immune responses induced by the heterologous regimen. These findings suggest that adenoviral priming may induce a lasting innate immune activation at the injection site, creating a strong local immune environment that enhances responses upon booster vaccination. This effect is more pronounced with heterologous mRNA boosting, suggesting that pre-conditioned innate activation contributes to the greater immunological synergy observed in heterologous vaccination.

### Heterologous vaccination enhances fibroblast-mediated inflammatory response and stromal activation

**Figure 6:**
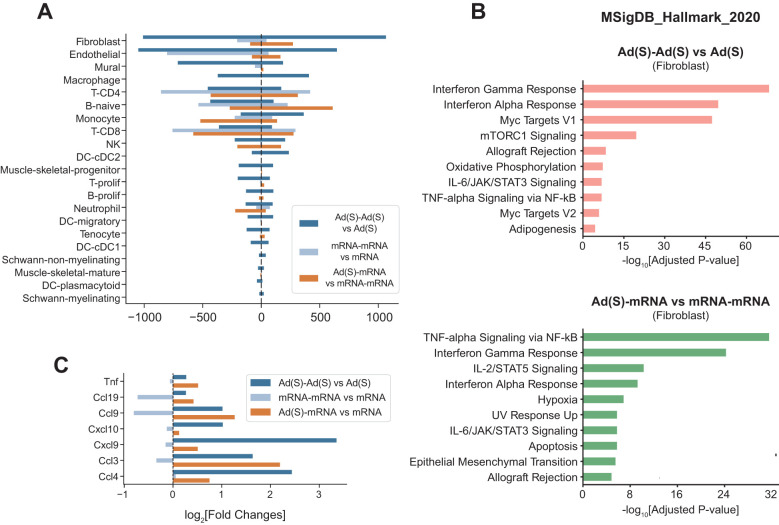
Heterologous vaccination amplifies fibroblast-driven inflammation and stromal activation. **(A)**, Number of differentially expressed genes (prime vs boost shot) across cell types in each injection condition. **(B)**, Gene set enrichment analysis using MSigDB Hallmark gene sets. Pathway enrichment analyses were conducted on the upregulated genes in fibroblasts: (top) adenoviral vector boost shot injection vs adenoviral vector prime shot, (bottom) cross vaccination vs boost shot injection of mRNA vaccine. **(C)**, Cytokine gene expression changes in fibroblasts in multiple comparison conditions. Indicated values are log2-fold changes of gene expressions in each comparison.

When comparing primary and boost vaccinations, fibroblasts exhibited the high spike mRNA detection and the most significant DEG changes among all cell types analyzed (**Figure 6**), prompting further investigation of their functional roles. Pathway enrichment analysis revealed significant upregulation of inflammatory pathways, majorly in interferon responses, in fibroblasts following the homologous adenoviral booster dose (**Figure 6**). Notably, DEGs in the heterologous mRNA booster dose were primarily linked to a stronger inflammatory response, characterized by enhanced TNF-alpha signaling and interferon responses, compared to the homologous mRNA booster vaccination.

Cytokine and chemokine expression analyses in fibroblasts revealed markedly increased chemokine expression after the adenoviral vector boost compared to the prime shot (**Figure 6**, dark blue bars). A similar pattern was observed in heterologous vaccination, where the mRNA vaccine boost after adenoviral vector priming induced significantly higher chemokine expression than homologous mRNA boosting (**Figure 6**, orange bars).

Taken together, these results establish fibroblasts as key mediators of booster-induced inflammatory responses, with heterologous vaccination more effectively amplifying both stromal activation and dendritic cell activity. These findings provide mechanistic insight into the enhanced immune responses observed in heterologous prime-boost regimens.

## Discussion

Our study highlights the immunological advantages of heterologous vaccination regimens, specifically, the combination of adenoviral and mRNA vaccines, in a controlled mouse model. Through a direct comparison of vaccine platforms, we demonstrated that heterologous vaccination yielded stronger humoral and cellular immune responses against the prototypical D614 virus and SARS-CoV-2 variants, particularly Delta and Omicron. These results are consistent with the existing clinical observations that a heterologous regimen can induce comparable or stronger humoral and/or cellular immune responses than both the homologous adenoviral vector regimen (46–52) and the homologous mRNA vaccine regimen (16, 47–51, 53, 54). Likewise, comparative effectiveness was found between heterologous adenoviral vectors and mRNA vaccines than homologous adenoviral vector vaccine (55), underscoring the benefit of using different vaccine types together to enhance protection against SARS-CoV-2 variants.

Our data corroborated that heterologous vaccination leads to significantly higher neutralizing antibody titers against the D614, Delta, and Omicron variants than homologous vaccination. This outcome may be due to the cross-stimulation of immune pathways, as adenovirus-vectored vaccines are prone to induce a strong T-cell response but are weak in generating neutralizing antibodies, and vice versa for mRNA vaccines. Additionally, efficient antibody class switching was observed in the heterologous group. The elevated IgG titers and balanced IgG2a/IgG1 ratio suggest that heterologous vaccination achieves an optimal Th1/Th2 balance, which is beneficial for sustained antibody production and memory formation (56). This balanced response may provide broader protection not only through neutralization but also through enhanced antibody-dependent cellular cytotoxicity, which is especially important in the context of evolving SARS-CoV-2 variants. Notably, while the homologous mRNA and heterologous vaccine regimens elicited comparable levels of spike-specific total IgG, the heterologous group exhibited significantly higher neutralizing antibody titers. This discrepancy suggests that the heterologous regimen may have induced greater expansion of the nAb population or superior affinity maturation. Recent studies have shown increased BCR clonal diversity and somatic hypermutation in heterologous vaccine recipients (54, 57, 58). Future work incorporating BCR repertoire and antibody quality assessments will be essential to confirm this mechanism.

Heterologous vaccination elicited robust T cell responses, as evidenced by the increased numbers of IFN-γ- and TNF-α-producing CD8 T cells. The magnitude of the CD8^+^ T cell response was greater than that observed with either homologous vaccine regimen, suggesting that combining the two vaccine platforms had a synergistic effect in enhancing the cellular immune response. This finding is crucial, as the CD8^+^ T cell response plays a critical role in eliminating virus-infected cells, which may reduce viral replication early in infection and provide broader protection against potential variants (59, 60). The ability of heterologous vaccination regimens to elicit such responses may be particularly beneficial for populations at higher risk, providing a more comprehensive and durable immune defense compared to single-platform regimens (4, 61, 62). However, as our analysis was limited to a single post-boost time point (day 21), the durability of the observed responses remains to be determined. Longitudinal studies tracking memory T cell subsets will be necessary to assess immune persistence.

To investigate how differences in the local tissue environment contribute to systemic immune responses, we performed single-cell profiling of the injection site. Single-cell transcriptomic analysis revealed distinct differences in the immune microenvironment at the injection site between the mRNA and adenoviral vaccines. The adenoviral vector vaccine induced fewer changes in cellular composition and lower level of spike transcripts at the injection site than the mRNA vaccine which prominently recruited monocytes, neutrophils, and CD8 T cells due to LNP component (30). These results align with the facts that ability of mRNA vaccines to initiate instant mRNA translation and to induce a robust early immune response driven by the localized adjuvant-like effect (63, 64), contrasting to the slower gene expression and antigen presentation of adenoviruses due to the time of delivering DNA to the cell nucleus (65). Regardless of the type of vaccine and regimen, the enrichment of spike transcripts was highest in fibroblasts, indicating that fibroblasts play a key role in the local production of antigens at the injection site.

Notably, the homologous adenoviral booster amplified innate immune responses more significantly while the homologous mRNA booster remained similar level, likely owing to trained immunity. This observation may explain the higher cellular and humoral response observed with the heterologous prime-boost regimen, where adenoviral vector vaccine predominantly induces cellular immunity and mRNA vaccine strongly induces humoral immunity (66). Furthermore, the synergy between the characteristics of adenoviral priming and mRNA boosting leads to nAb titers. The slow and sustained antigen release from the adenoviral priming dose allows for immune responses not only to dominant epitopes but also to subdominant one (67). This is complemented by the potent cellular and humoral immune activation triggered by the mRNA booster, emphasizing the complementary mechanisms of these two platforms.

Significant influence on these immune responses seems to be mediated by fibroblasts, which act as key contributors to injection site inflammation. Fibroblasts exhibit upregulation of inflammatory pathways and chemokine production, particularly following the homologous adenoviral boosting and heterologous vaccination. Given that fibroblast are the primary targets of adenoviral vectors at the injection site (68), our data suggest that adenoviral priming induces transcriptional changes in these cells, potentially imprinting an innate memory-like state. These imprinting may enhance chemokine responses and facilitate the recruitment and activation of other immune cells during boost vaccination, regardless of whether the booster is a homologous adenoviral vector or a heterologous mRNA vaccine. The ability of fibroblasts to establish an inflammatory milieu and potentially adopt an innate memory-like state may provide a mechanistic basis for the superior immune response observed with heterologous regimens.

Beyond their role in initiating local inflammation, fibroblast activation may influence broader immune organization at the tissue level. Fibroblasts are increasingly recognized as active regulators of immune responses across diverse tissue environment, where they contribute to immune cell positioning, chemokine signaling, and the structural organization of local immune niches (69, 70). Under inflammatory conditions, fibroblasts can acquire stromal organizer–like properties, guiding leukocyte clustering and supporting antigen presentation (71). In our study, the elevated expression of Ccl3, Ccl9, and Ccl19 in injection-site fibroblasts following adenoviral priming and mRNA boosting suggests that these cells contribute to the recruitment and coordination of innate and adaptive immune responses. Rather than acting as passive bystanders, fibroblasts may help shape a tissue microenvironment that promotes effective immune activation in the context of heterologous vaccination. These findings support a model in which stromal priming by the initial vaccine dose enhances the immunological impact of subsequent boosting.

Although our single-cell analysis does not fully account for the systemic enhancement of humoral or CD8^+^ T cell responses, it provides important mechanistic context. Recent findings by Kim et al. (30) demonstrated that fibroblast-derived IFN-β at the injection site is essential for activating dendritic cells and initiating vaccine-induced T cell responses. These results support the biological plausibility of our model, in which stromal priming through adenoviral vaccination alters the tissue microenvironment and enhances responsiveness to mRNA boosting.

In conclusion, this study provides compelling evidence that heterologous vaccination, an adenoviral prime followed by an mRNA boost, elicits stronger and more comprehensive immune responses than homologous regimens. The observed combination of robust humoral immunity, balanced Th1/Th2 responses, and enhanced CD8^+^ T cell activation highlights the immunological advantages of platform mixing in overcoming the challenges posed by emerging SARS-CoV-2 variants. Our findings are consistent with prior clinical observations and offer mechanistic insight into how heterologous prime-boost strategies can optimize immune activation by shaping the local stromal and cellular environment at the injection site.

Taken together, these results lay a foundation for future immunization strategies that harness the complementary strengths of different vaccine platforms to enhance both the magnitude and breadth of protective immunity. Although this study was not designed to assess direct protective efficacy or to functionally validate all proposed mechanisms, future studies incorporating *in vivo* protection models and *in vitro* validation will be essential to further elucidate these pathways and evaluate their translational potential.

## Data Availability

Raw single-cell RNA sequencing data generated in this study were deposited in the Gene Expression Omnibus database under the accession code GSE282488.

## Supplementary material

Supplementary Figure 1 Kinetics of severe acute respiratory syndrome coronavirus 2 (SARS-CoV-2) spike specific binding antibody responses against total immunoglobulin M (IgM).

Supplementary Figure 2
**(A)**, Flow cytometry analysis of intracellular cytokines IFN-γ, TNF-α double positive CD3^+^CD8^+^ T cells. Antigen-specific CD8 T cells were obtained from mouse spleen samples on day 42 post-vaccination. **(B, C)**, **(B)** IFN-γ and **(C)** TNF-α positive CD3^+^CD4^+^ cells were stimulated with D614, Delta, and BA.5 spike glycoprotein peptide pool. Antigen-specific CD4 T cells were obtained from mouse spleen samples on day 42 post-vaccination. **(D)**, Flow cytometry analysis of intracellular cytokines IFN-γ, TNF-α double positive CD3^+^CD4^+^ T cells.

## References

[1] Ho TC Chen YA Chan HP Chang CC Chuang KP Lee CH. The effects of heterologous immunization with prime-boost COVID-19 vaccination against SARS-CoV-2. Vaccines (Basel). (2021) 9(10):1163. doi: 10.3390/vaccines9101163 34696271 PMC8537265

[2] Garg I Sheikh AB Pal S Shekhar R. Mix-and-match COVID-19 vaccinations (Heterologous boost): A review. Infect Dis Rep. (2022) 14:537–46. doi: 10.3390/idr14040057 PMC9326526 35893476

[3] Costa Clemens SA Weckx L Clemens R Almeida Mendes AV Ramos Souza A Silveira MBV. Heterologous versus homologous COVID-19 booster vaccination in previous recipients of two doses of CoronaVac COVID-19 vaccine in Brazil (RHH-001): a phase 4, non-inferiority, single blind, randomised study. Lancet. (2022) 399:521–9. doi: 10.1016/S0140-6736(22)00094-0 PMC8782575 35074136

[4] Au WY Cheung PP. Effectiveness of heterologous and homologous covid-19 vaccine regimens: living systematic review with network meta-analysis. Bmj. (2022) 377:e069989. doi: 10.1136/bmj-2022-069989 35640925 PMC9724446

[5] Cohen LE Spiro DJ Viboud C. Projecting the SARS-CoV-2 transition from pandemicity to endemicity: Epidemiological and immunological considerations. PLoS Pathogens. (2022) 18:e1010591. doi: 10.1371/journal.ppat.1010591 35771775 PMC9246171

[6] Malik JA Ahmed S Mir A Shinde M Bender O Alshammari F. The SARS-CoV-2 mutations versus vaccine effectiveness: New opportunities to new challenges. J Infection Public Health. (2022) 15:228–40. doi: 10.1016/j.jiph.2021.12.014 PMC8730674 35042059

[7] Aleem A Akbar Samad AB Vaqar S. Emerging Variants of SARS-CoV-2 and Novel Therapeutics Against Coronavirus (COVID-19). Treasure Island (FL: StatPearls Publishing (2023). 34033342

[8] World Health Organization (WHO). Currently circulating variants of interest (VOIs) and variants under monitoring (VUMs). World Health Organization. (2025). Available online at: https://www.who.int/activities/tracking-SARS-CoV-2-variants.

[9] Nguyen TT Quach THT Tran TM Phuoc HN Nguyen HT Vo TK. Reactogenicity and immunogenicity of heterologous prime-boost immunization with COVID-19 vaccine. BioMed Pharmacother. (2022) 147:112650. doi: 10.1016/j.biopha.2022.112650 35066301 PMC8767802

[10] Sapkota B Saud B Shrestha R Al-Fahad D Sah R Shrestha S. Heterologous prime-boost strategies for COVID-19 vaccines. J Travel Med. (2022) 29(3):taab191. doi: 10.1093/jtm/taab191 34918097 PMC8754745

[11] Choudhary OP Priyanka Ahmed JQ Mohammed TA Singh I Rodriguez-Morales AJ. Heterologous prime-boost vaccination against COVID-19: is it safe and reliable? Hum Vaccin Immunother. (2021) 17:5135–8. doi: 10.1080/21645515.2021.2007015 PMC8726007 34898381

[12] Ai J Zhang H Zhang Y Lin K Zhang Y Wu J. Omicron variant showed lower neutralizing sensitivity than other SARS-CoV-2 variants to immune sera elicited by vaccines after boost. Emerg Microbes Infect. (2022) 11:337–43. doi: 10.1080/22221751.2021.2022440 PMC8788341 34935594

[13] Bae S Ko JH Choi JY Park WJ Lim SY Ahn JY. Heterologous ChAdOx1 and Bnt162b2 vaccination induces strong neutralizing antibody responses against SARS-CoV-2 including delta variant with tolerable reactogenicity. Clin Microbiol Infect. (2022) 28:1390.e1–.e7. doi: 10.1016/j.cmi.2022.04.019 PMC9117169 35598855

[14] Liu X Li Y Wang Z Cao S Huang W Yuan L. Safety and superior immunogenicity of heterologous boosting with an RBD-based SARS-CoV-2 mRNA vaccine in Chinese adults. Cell Res. (2022) 32:777–80. doi: 10.1038/s41422-022-00681-3 PMC9197092 35701541

[15] Cheng H Peng Z Si S Alifu X Zhou H Chi P. Immunogenicity and safety of homologous and heterologous prime-boost immunization with COVID-19 vaccine: systematic review and meta-analysis. Vaccines (Basel). (2022) 10(1):53. doi: 10.3390/vaccines10050798 PMC9142990 35632554

[16] Vogel E Kocher K Priller A Cheng CC Steininger P Liao BH. Dynamics of humoral and cellular immune responses after homologous and heterologous SARS-CoV-2 vaccination with ChAdOx1 nCoV-19 and BNT162b2. EBioMedicine. (2022) 85:104294. doi: 10.1016/j.ebiom.2022.104294 36206622 PMC9530590

[17] He Q Mao Q An C Zhang J Gao F Bian L. Heterologous prime-boost: breaking the protective immune response bottleneck of COVID-19 vaccine candidates. Emerg Microbes Infect. (2021) 10:629–37. doi: 10.1080/22221751.2021.1902245 PMC8009122 33691606

[18] Westrop SJ Whitaker HJ Powell AA Power L Whillock C Campbell H. Real-world data on immune responses following heterologous prime-boost COVID-19 vaccination schedule with Pfizer and AstraZeneca vaccines in England. J Infect. (2022) 84:692–700. doi: 10.1016/j.jinf.2022.01.038 35131335 PMC8815191

[19] Atmar RL Lyke KE Deming ME Jackson LA Branche AR El Sahly HM. Homologous and heterologous covid-19 booster vaccinations. N Engl J Med. (2022) 386:1046–57. doi: 10.1056/NEJMoa2116414 PMC8820244 35081293

[20] Peng D Zhao T Hong W Fu M He C Chen L. Heterologous vaccination with subunit protein vaccine induces a superior neutralizing capacity against BA.4/5-included SARS-CoV-2 variants than homologous vaccination of mRNA vaccine. MedComm (2020). (2023) 4:e238. doi: 10.1002/mco2.238 36911160 PMC10000276

[21] Hong W Lei H Peng D Huang Y He C Yang J. A chimeric adenovirus-vectored vaccine based on Beta spike and Delta RBD confers a broad-spectrum neutralization against Omicron-included SARS-CoV-2 variants. MedComm (2020). (2024) 5:e539. doi: 10.1002/mco2.v5.5 38680520 PMC11055958

[22] Murphy DM Cox DJ Connolly SA Breen EP Brugman AA Phelan JJ. Trained immunity is induced in humans after immunization with an adenoviral vector COVID-19 vaccine. J Clin Invest. (2023) 133(2):e162581. doi: 10.1172/JCI162581 36282571 PMC9843058

[23] Netea MG Joosten LA. Beyond adaptive immunity: induction of trained immunity by COVID-19 adenoviral vaccines. J Clin Invest. (2023) 133(2):e166467. doi: 10.1172/JCI166467 36647822 PMC9843054

[24] Marín-Hernández D Nixon DF Hupert N. Heterologous vaccine interventions: boosting immunity against future pandemics. Mol Medicine. (2021) 27:54. doi: 10.1186/s10020-021-00317-z PMC8165337 34058986

[25] Netea MG Joosten LA Latz E Mills KH Natoli G Stunnenberg HG. Trained immunity: A program of innate immune memory in health and disease. Science. (2016) 352:aaf1098. doi: 10.1126/science.aaf1098 27102489 PMC5087274

[26] Coughlan L. Factors which contribute to the immunogenicity of non-replicating adenoviral vectored vaccines. Front Immunol. (2020) 11:909. doi: 10.3389/fimmu.2020.00909 32508823 PMC7248264

[27] Palgen JL Feraoun Y Dzangué-Tchoupou G Joly C Martinon F Le Grand R. Optimize prime/boost vaccine strategies: trained immunity as a new player in the game. Front Immunol. (2021) 12:612747. doi: 10.3389/fimmu.2021.612747 33763063 PMC7982481

[28] Namgaladze D Brüne B. Rapid glycolytic activation accompanying innate immune responses: mechanisms and function. Front Immunol. (2023) 14:1180488. doi: 10.3389/fimmu.2023.1180488 37153593 PMC10158531

[29] López-Collazo E Del Fresno C. Endotoxin tolerance and trained immunity: breaking down immunological memory barriers. Front Immunol. (2024) 15:1393283. doi: 10.3389/fimmu.2024.1393283 38742111 PMC11089161

[30] Kim S Jeon JH Kim M Lee Y Hwang Y-H Park M. Innate immune responses against mRNA vaccine promote cellular immunity through IFN-β at the injection site. Nat Communications. (2024) 15:7226. doi: 10.1038/s41467-024-51411-9 PMC11349762 39191748

[31] Shin SP Shin KS Lee JM Jung IK Koo J Lee SW. The chimeric adenovirus (Ad5/35) expressing engineered spike protein confers immunity against SARS-CoV-2 in mice and non-human primates. Vaccines (Basel). (2022) 10(5):712. doi: 10.3390/vaccines10050712 35632468 PMC9147121

[32] Wolf FA Angerer P Theis FJ. SCANPY: large-scale single-cell gene expression data analysis. Genome Biol. (2018) 19:15. doi: 10.1186/s13059-017-1382-0 29409532 PMC5802054

[33] Wolock SL Lopez R Klein AM. Scrublet: computational identification of cell doublets in single-cell transcriptomic data. Cell Syst. (2019) 8:281–91.e9. doi: 10.1016/j.cels.2018.11.005 30954476 PMC6625319

[34] Polański K Young MD Miao Z Meyer KB Teichmann SA Park JE. BBKNN: fast batch alignment of single cell transcriptomes. Bioinformatics. (2020) 36:964–5. doi: 10.1093/bioinformatics/btz625 PMC9883685 31400197

[35] Büttner M Ostner J Müller CL Theis FJ Schubert B. scCODA is a Bayesian model for compositional single-cell data analysis. Nat Commun. (2021) 12:6876. doi: 10.1038/s41467-021-27150-6 34824236 PMC8616929

[36] Fang Z Liu X Peltz G. GSEApy: a comprehensive package for performing gene set enrichment analysis in Python. Bioinformatics. (2023) 39(1):btac757. doi: 10.1093/bioinformatics/btac757 36426870 PMC9805564

[37] Liberzon A Birger C Thorvaldsdóttir H Ghandi M Mesirov JP Tamayo P. The Molecular Signatures Database (MSigDB) hallmark gene set collection. Cell Syst. (2015) 1:417–25. doi: 10.1016/j.cels.2015.12.004 PMC4707969 26771021

[38] Zhang Z Mateus J Coelho CH Dan JM Moderbacher CR Gálvez RI. Humoral and cellular immune memory to four COVID-19 vaccines. Cell. (2022) 185:2434–51.e17. doi: 10.1016/j.cell.2022.05.022 35764089 PMC9135677

[39] Sheetikov SA Khmelevskaya AA Zornikova KV Zvyagin IV Shomuradova AS Serdyuk YV. Clonal structure and the specificity of vaccine-induced T cell response to SARS-CoV-2 Spike protein. Front Immunol. (2024) 15:1369436. doi: 10.3389/fimmu.2024.1369436 38629062 PMC11018901

[40] Wu Y Zhang H Meng L Li F Yu C. Comparison of immune responses elicited by SARS-CoV-2 mRNA and recombinant protein vaccine candidates. Front Immunol. (2022) 13:906457. doi: 10.3389/fimmu.2022.906457 35663946 PMC9161160

[41] Chung NH Chen YC Yang SJ Lin YC Dou HY Hui-Ching Wang L. Induction of Th1 and Th2 in the protection against SARS-CoV-2 through mucosal delivery of an adenovirus vaccine expressing an engineered spike protein. Vaccine. (2022) 40:574–86. doi: 10.1016/j.vaccine.2021.12.024 PMC8677488 34952759

[42] Li C Lee A Grigoryan L Arunachalam PS Scott MKD Trisal M. Mechanisms of innate and adaptive immunity to the Pfizer-BioNTech BNT162b2 vaccine. Nat Immunol. (2022) 23:543–55. doi: 10.1038/s41590-022-01163-9 PMC8989677 35288714

[43] Shen CF Yen CL Fu YC Cheng CM Shen TC Chang PD. Innate immune responses of vaccinees determine early neutralizing antibody production after chAdOx1nCoV-19 vaccination. Front Immunol. (2022) 13:807454. doi: 10.3389/fimmu.2022.807454 35145520 PMC8822242

[44] Föhse K Geckin B Zoodsma M Kilic G Liu Z Röring RJ. The impact of BNT162b2 mRNA vaccine on adaptive and innate immune responses. Clin Immunol. (2023) 255:109762. doi: 10.1016/j.clim.2023.109762 37673225

[45] Iwasaki A Medzhitov R. Control of adaptive immunity by the innate immune system. Nat Immunol. (2015) 16:343–53. doi: 10.1038/ni.3123 PMC4507498 25789684

[46] Barros-Martins J Hammerschmidt SI Cossmann A Odak I Stankov MV Morillas Ramos G. Immune responses against SARS-CoV-2 variants after heterologous and homologous ChAdOx1 nCoV-19/BNT162b2 vaccination. Nat Medicine. (2021) 27:1525–9. doi: 10.1038/s41591-021-01449-9 PMC8440184 34262158

[47] Bauswein M Peterhoff D Plentz A Hiergeist A Wagner R Gessner A. Increased neutralization of SARS-CoV-2 Delta variant after heterologous ChAdOx1 nCoV-19/BNT162b2 versus homologous BNT162b2 vaccination. iScience. (2022) 25:103694. doi: 10.1016/j.isci.2021.103694 35013723 PMC8730691

[48] Benning L Töllner M Hidmark A Schaier M Nusshag C Kälble F. Heterologous ChAdOx1 nCoV-19/BNT162b2 Prime-Boost Vaccination Induces Strong Humoral Responses among Health Care Workers. Vaccines (Basel). (2021) 9(8):857. doi: 10.3390/vaccines9080857 34451982 PMC8402499

[49] Firinu D Perra A Campagna M Littera R Meloni F Sedda F. Evaluation of antibody response to heterologous prime-boost vaccination with chAdOx1 nCoV-19 and BNT162b2: an observational study. Vaccines (Basel). (2021) 9(12):1478. doi: 10.3390/vaccines9121478 34960224 PMC8704060

[50] Hillus D Schwarz T Tober-Lau P Vanshylla K Hastor H Thibeault C. Safety, reactogenicity, and immunogenicity of homologous and heterologous prime-boost immunisation with ChAdOx1 nCoV-19 and BNT162b2: a prospective cohort study. Lancet Respir Med. (2021) 9:1255–65. doi: 10.1016/S2213-2600(21)00357-X PMC8360702 34391547

[51] Tenbusch M Schumacher S Vogel E Priller A Held J Steininger P. Heterologous prime-boost vaccination with ChAdOx1 nCoV-19 and BNT162b2. Lancet Infect Dis. (2021) 21:1212–3. doi: 10.1016/S1473-3099(21)00420-5 PMC8321428 34332707

[52] González S Olszevicki S Gaiano A Salazar M Regairaz L Varela Baino AN. Protection of homologous and heterologous boosters after primary schemes of rAd26-rAd5, ChAdOx1 nCoV-19 and BBIBP-CorV during the omicron outbreak in adults of 50 years and older in Argentina: a test-negative case-control study. Lancet Reg Health Am. (2023) 27:100607. doi: 10.1016/j.lana.2023.100607 37808936 PMC10558771

[53] Groß R Zanoni M Seidel A Conzelmann C Gilg A Krnavek D. Heterologous ChAdOx1 nCoV-19 and BNT162b2 prime-boost vaccination elicits potent neutralizing antibody responses and T cell reactivity against prevalent SARS-CoV-2 variants. EBioMedicine. (2022) 75:103761. doi: 10.1016/j.ebiom.2021.103761 34929493 PMC8682749

[54] Lee HK Go J Sung H Kim SW Walter M Knabl L. Heterologous ChAdOx1-BNT162b2 vaccination in Korean cohort induces robust immune and antibody responses that includes Omicron. iScience. (2022) 25:104473. doi: 10.1016/j.isci.2022.104473 35637788 PMC9132682

[55] Hermosilla E Coma E Xie J Feng S Cabezas C Méndez-Boo L. Comparative effectiveness and safety of homologous two-dose ChAdOx1 versus heterologous vaccination with ChAdOx1 and BNT162b2. Nat Communications. (2022) 13:1639. doi: 10.1038/s41467-022-29301-9 PMC8943099 35322045

[56] Smith KM Pottage L Thomas ER Leishman AJ Doig TN Xu D. Th1 and Th2 CD4+ T cells provide help for B cell clonal expansion and antibody synthesis in a similar manner *in vivo* . J Immunol. (2000) 165:3136–44. doi: 10.4049/jimmunol.165.6.3136 10975827

[57] Zhao W Zhao H Huang B Zhao T Wang L Zhang J. Unravelling the enhanced vaccine immunity by heterologous KCONVAC/Ad5-nCoV COVID-19 vaccination. Signal Transduct Target Ther. (2022) 7:210. doi: 10.1038/s41392-022-01079-8 35787610 PMC9251036

[58] Song Y Wang J Yang Z He Q Bao C Xie Y. Heterologous booster vaccination enhances antibody responses to SARS-CoV-2 by improving Tfh function and increasing B-cell clonotype SHM frequency. Front Immunol. (2024) 15:1406138. doi: 10.3389/fimmu.2024.1406138 38975334 PMC11224535

[59] Liu J Yu J McMahan K Jacob-Dolan C He X Giffin V. CD8 T cells contribute to vaccine protection against SARS-CoV-2 in macaques. Sci Immunol. (2022) 7:eabq7647. doi: 10.1126/sciimmunol.abq7647 35943359 PMC9407944

[60] Vieira VA Herbert N Cromhout G Adland E Goulder P. Role of early life cytotoxic T lymphocyte and natural killer cell immunity in paediatric HIV cure/remission in the anti-retroviral therapy era. Front Immunol. (2022) 13:886562. doi: 10.3389/fimmu.2022.886562 35634290 PMC9130627

[61] Rizvi NB Bibi M Rana MZ Zaffar S Farooq H. Comparison of antibody responses of heterologous and homologous Covid-19 booster vaccination: an observational study. Front Immunol. (2024) 15:1448408. doi: 10.3389/fimmu.2024.1448408 39606247 PMC11598339

[62] Elliott T Cheeseman HM Evans AB Day S McFarlane LR O’Hara J. Enhanced immune responses following heterologous vaccination with self-amplifying RNA and mRNA COVID-19 vaccines. PLoS Pathog. (2022) 18:e1010885. doi: 10.1371/journal.ppat.1010885 36194628 PMC9565686

[63] Alameh MG Tombácz I Bettini E Lederer K Sittplangkoon C Wilmore JR. Lipid nanoparticles enhance the efficacy of mRNA and protein subunit vaccines by inducing robust T follicular helper cell and humoral responses. Immunity. (2021) 54:2877–92.e7. doi: 10.1016/j.immuni.2021.11.001 34852217 PMC8566475

[64] Ndeupen S Qin Z Jacobsen S Bouteau A Estanbouli H Igyártó BZ. The mRNA-LNP platform’s lipid nanoparticle component used in preclinical vaccine studies is highly inflammatory. iScience. (2021) 24:103479. doi: 10.1016/j.isci.2021.103479 34841223 PMC8604799

[65] Pardi N Hogan MJ Porter FW Weissman D. mRNA vaccines — a new era in vaccinology. Nat Rev Drug Discovery. (2018) 17:261–79. doi: 10.1038/nrd.2017.243 PMC5906799 29326426

[66] Provine NM Klenerman P. Adenovirus vector and mRNA vaccines: Mechanisms regulating their immunogenicity. Eur J Immunol. (2023) 53:e2250022. doi: 10.1002/eji.202250022 36330560 PMC9877955

[67] Rollier CS Hill AVS Reyes-Sandoval A. Influence of adenovirus and MVA vaccines on the breadth and hierarchy of T cell responses. Vaccine. (2016) 34:4470–4. doi: 10.1016/j.vaccine.2016.07.050 PMC5009894 27484012

[68] Cupovic J Ring SS Onder L Colston JM Lütge M Cheng H-W. Adenovirus vector vaccination reprograms pulmonary fibroblastic niches to support protective inflating memory CD8+ T cells. Nat Immunol. (2021) 22:1042–51. doi: 10.1038/s41590-021-00969-3 PMC7611414 34267375

[69] Buechler MB Pradhan RN Krishnamurty AT Cox C Calviello AK Wang AW. Cross-tissue organization of the fibroblast lineage. Nature. (2021) 593:575–9. doi: 10.1038/s41586-021-03549-5 33981032

[70] Onder L Papadopoulou C Lütge A Cheng HW Lütge M Perez-Shibayama C. Fibroblastic reticular cells generate protective intratumoral T cell environments in lung cancer. Cell. (2025) 188:430–46.e20. doi: 10.1016/j.cell.2024.10.042 39566495

[71] Barone F Gardner DH Nayar S Steinthal N Buckley CD Luther SA. Stromal fibroblasts in tertiary lymphoid structures: A novel target in chronic inflammation. Front Immunol. (2016) 7:477. doi: 10.3389/fimmu.2016.00477 27877173 PMC5100680

