# Supplementary material for: Heterologous prime-boost vaccination drives stromal activation and adaptive immunity against SARS-CoV-2 variants
Source: Front Immunol. 2025 May 28;16:1597417. doi: 10.3389/fimmu.2025.1597417 (PMC12151836; doi:10.3389/fimmu.2025.1597417)
Supplement: Supplementary Figure 1 — Kinetics of severe acute respiratory syndrome coronavirus 2 (SARS-CoV-2) spike specific binding antibody responses against total immunoglobulin M (IgM). [file Image1.pdf]

Supplementary Figure 1

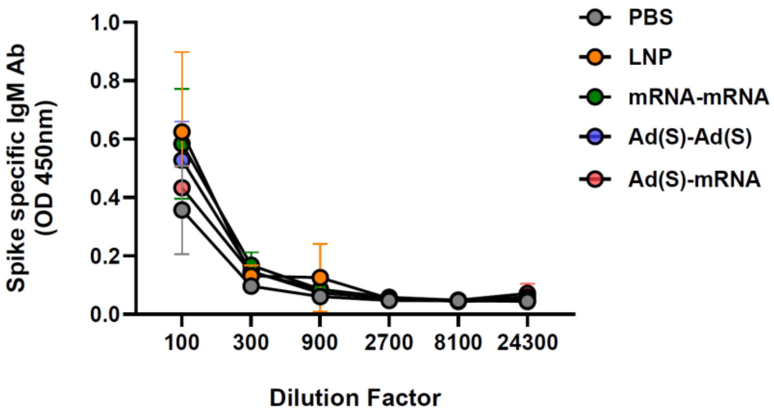

Supplementary Figure 1. Kinetics of severe acute respiratory syndrome coronavirus 2 (SARS-CoV-2) spike specific binding antibody responses against total immunoglobulin M (IgM).
