# Supplementary material for: Heterologous prime-boost vaccination drives stromal activation and adaptive immunity against SARS-CoV-2 variants
Source: Front Immunol. 2025 May 28;16:1597417. doi: 10.3389/fimmu.2025.1597417 (PMC12151836; doi:10.3389/fimmu.2025.1597417)
Supplement: Supplementary Figure 2 — (A), Flow cytometry analysis of intracellular cytokines IFN-γ, TNF-α double positive CD3+CD8+ T cells. Antigen-specific CD8 T cells were obtained from mouse spleen samples on day 42 post-vaccination. (B, C), (B) IFN-γ and (C) TNF-α positive CD3+CD4+ cells were stimulated with D614, Delta, and BA.5 spike glycoprotein peptide pool. Antigen-specific CD4 T cells were obtained from mouse spleen samples on day 42 post-vaccination. (D), Flow cytometry analysis of intracellular cytokines IFN-γ, TNF-α double positive CD3+CD4+ T cells. [file Image2.pdf]

Supplementary Figure 2

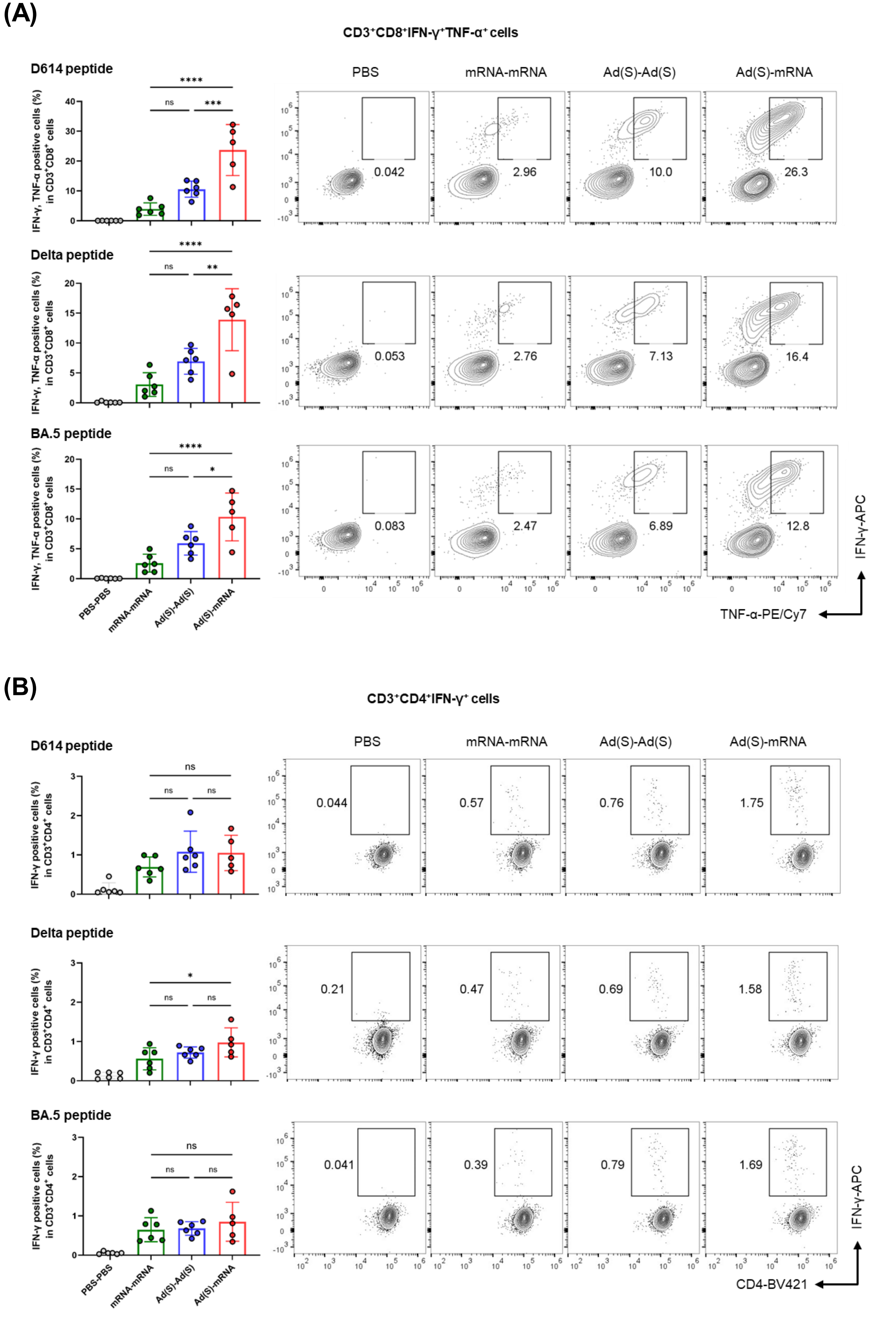

Supplementary Figure 2

(C)

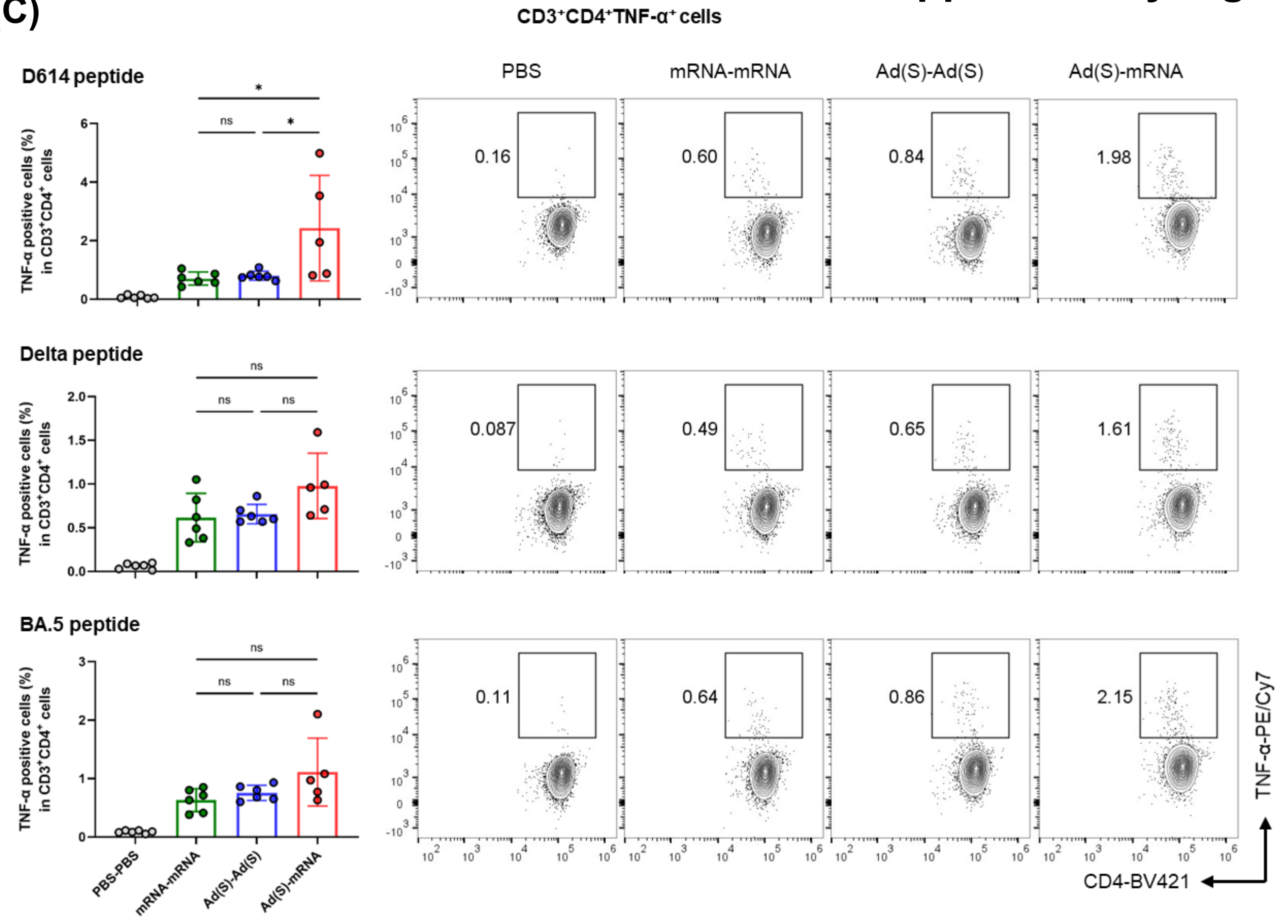

(D)

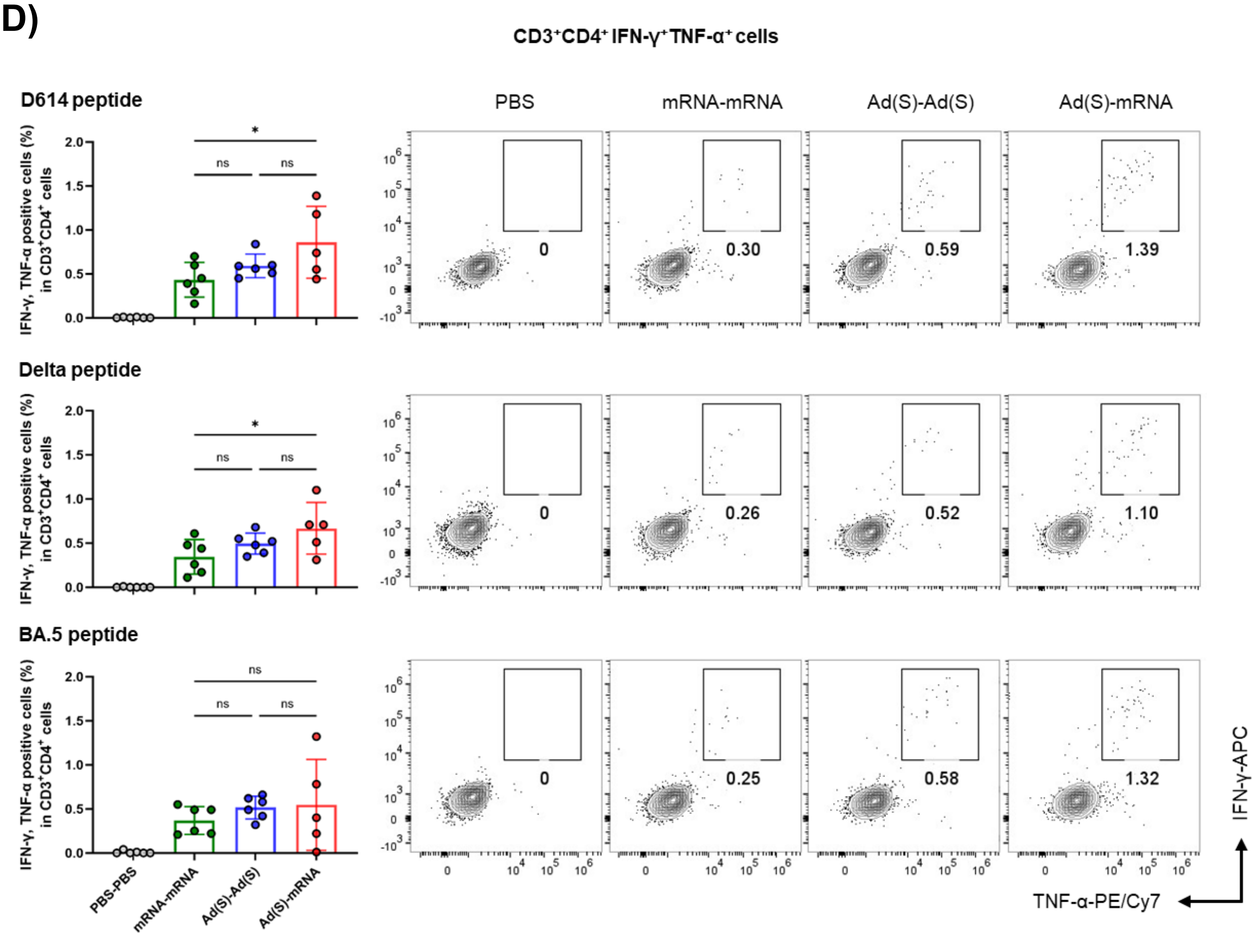

Supplementary Figure 2

A, Flow cytometry analysis of intracellular cytokines IFN-γ, TNF-α double positive $CD3^+CD8^+$ T cells. Antigen-specific CD8 T cells were obtained from mouse spleen samples on day 42 post-vaccination. **B,C, (B)** IFN-γ and **(C)** TNF-α positive $CD3^+CD4^+$ cells were stimulated with D614, Delta, and BA.5 spike glycoprotein peptide pool. Antigen-specific CD4 T cells were obtained from mouse spleen samples on day 42 post-vaccination. **D,** Flow cytometry analysis of intracellular cytokines IFN-γ, TNF-α double positive $CD3^+CD4^+$ T cells.
